# Physiological Impacts and Medical Applications of the Inspiratory Gasp Vascular Reflex

**DOI:** 10.7759/cureus.105709

**Published:** 2026-03-23

**Authors:** Harvey N Mayrovitz

**Affiliations:** 1 Medical Education, Nova Southeastern University Dr. Kiran C. Patel College of Allopathic Medicine, Davie, USA

**Keywords:** diabetes, inspiratory gasp, inspiratory gasp test, laser doppler, ldf, neurovascular dysfunction, peripheral neuropathy, ppg, skin blood flow, sympathetic dysfunction

## Abstract

Measurement of skin blood flow in the hand, foot, finger, or toe reveals a transient decrease in response to a rapid, deep inspiration, a phenomenon termed the inspiratory gasp vascular reflex (IGVR). The use of the IGVR as a test in future research and clinical applications requires knowledge of the method, its prior applications, its limitations and advantages, and the identification of clinically relevant knowledge gaps. The goal of this review is to consolidate and critically evaluate the findings of early workers, as well as the continuum of evolving prior knowledge regarding key aspects of this reflex. This report is derived from information obtained from an analysis of published material secured via literature searches of four major electronic databases and, in part, based on the author’s experience with the method. The literature search covered all years up to 2026 and used the primary search string: (“inspiratory gasp” OR “inspiratory gasp vascular reflex” OR “inspiratory gasp reflex”). Studies considered relevant for inclusion were those that evaluated the IGVR in healthy persons or persons with underlying conditions. The overriding evidence from the literature indicates that the IGVR can provide useful information about the status of peripheral neural function. However, its application and interpretation may be deceptively simple. The measurement methodology is straightforward, using either laser Doppler flowmetry or photoplethysmography to measure changes induced by one or more inspiratory gasps. However, measurement conditions contribute to variability in response magnitude, making it difficult to establish “normal” ranges. It is concluded that the most useful application of the IGVR test is to assess changes in peripheral neurovascular function in individuals following interventions or treatments that target such functions or to track longitudinal changes. Its use in discriminating between patient groups with various peripheral neurovascular dysfunctions and controls is limited by the absence of suitable normal reference ranges and standardized measurement protocols. However, when confounding factors are considered and group differences are sufficiently large, the IGVR can be a useful test.

## Introduction and background

Inspiratory gasp vascular reflex (IGVR)

**Figure 1:**
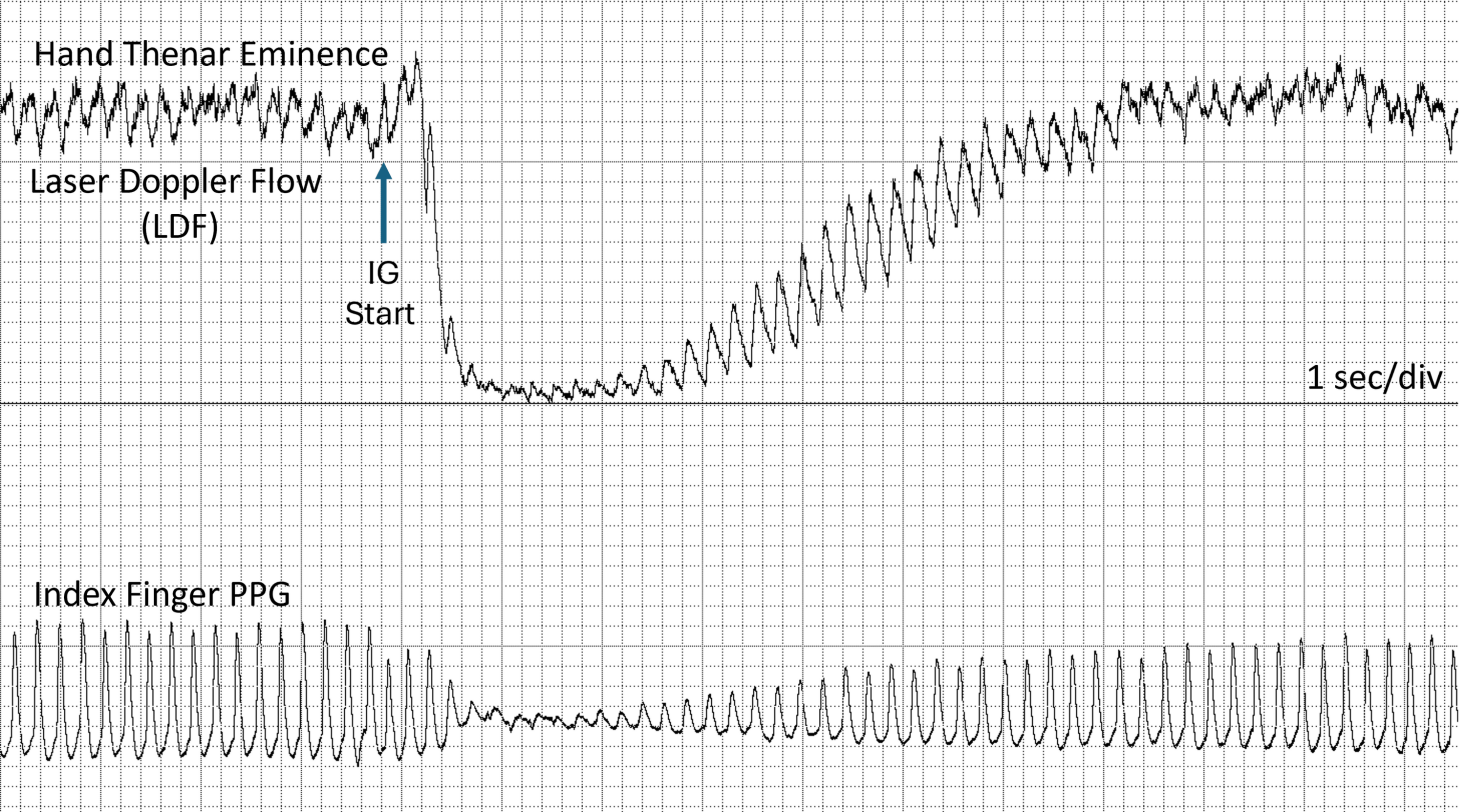
Inspiratory gasp reflex Skin blood flow is measured on the left hand’s thenar eminence using LDF, and the pulsatile PPG signal is measured on the index finger of the same hand. At the arrow, the subject (HNM) takes a deep IG and then relaxes approximately two seconds later. The reflex is associated with precipitous, transient decreases in LDF and PPG, followed by recovery toward baseline values. The rhythmic pulsations in LDF and PPG are due to the heartbeat. The overall duration of the reflex to recovery in this image is approximately 34 seconds, with the interval of near-zero LDF at approximately 10 seconds. IG, inspiratory gasp; LDF, laser Doppler flowmetry; PPG, photoplethysmography This previously unpublished figure is provided courtesy of Harvey N. Mayrovitz.

When blood flow is measured in the skin of the hand, foot, finger, or toe, and the individual takes a rapid, deep inspiration, the blood flow transiently decreases. This vascular change, the IGVR, is illustrated in Figure 1. In this figure, the skin blood flow is measured on the hand thenar eminence using the laser Doppler flowmetry (LDF) method [1,2], while at the same time, the photoplethysmography (PPG) signal, which is related to the cardiac-produced volume pulse, is measured on the index finger of the same hand [3,4]. In response to the approximately two-second inspiratory gasp (IG), both LDF and PPG signals initially decrease precipitously, then recover toward baseline.

**Figure 2:**
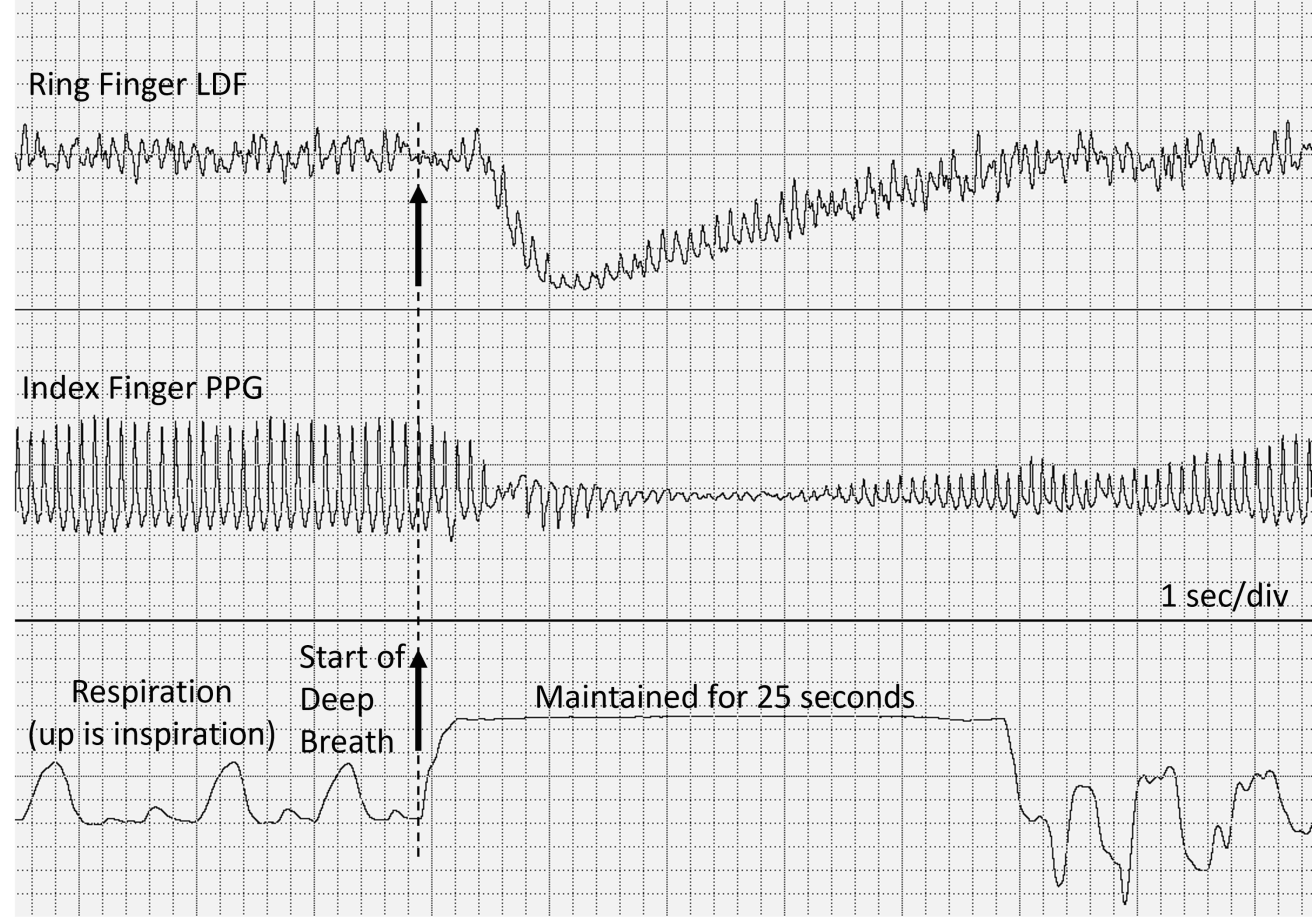
IG with maintained breath hold Skin blood flow is measured on the ring finger of the left hand with LDF, and the PPG signal is measured on the index finger of the same hand. At the arrow, the subject takes a rapid, deep inspiration and then maintains it for approximately 25 seconds before exhalation. The vascular responses are similar to those produced by a rapid inspiratory gasp and rapid release, as illustrated in Figure 1. IG, inspiratory gasp; LDF, laser Doppler flowmetry; PPG, photoplethysmography This previously unpublished figure is provided courtesy of Harvey N. Mayrovitz.

A similar vascular response can be observed following an IG, with maintained inspiration for about 25 seconds after an initial rapid and deep inspiration, as illustrated in Figure 2 [5]. Such a vasomotor reflex triggered by a rapid, deep inspiration causes arteriolar vasoconstriction, and this transient decrease in peripheral blood flow is common in healthy people.

Early findings

The effect of a rapid and deep inspiration on the peripheral pulse was first described by the French researchers Binet and Soller in 1895 [6]. However, quantitative information emerged in an early 1936 report by Bolton et al. [7], and later in 1939 by Mulinos and Shulman [8] and in 1948 by Gilliatt [9]. To study these phenomena, early researchers employed ingenuity and a variety of methods to experimentally assess the IGVR using various forms of plethysmography. This required elaborate experimental arrangements, including sealing the hand, foot, or individual digits in glass containers, and complex methods to determine volume changes using novel plethysmography techniques. These and other issues significantly limited the practical use of their important findings at the time. Over time, clinical applications have been undertaken and reported for patients with various conditions. However, because aspects of this reflex can now, and in the future, be measured with relative ease using widely available LDF and PPG devices, there is an opportunity to reconsider this reflex and to potentially develop new clinical applications.

Study purpose

If this method is to be considered in evolving research or clinical investigations, it is important to establish a solid foundation for its use. This requires knowledge of the method and its prior clinical applications, its limitations and advantages, and the identification of clinically relevant knowledge gaps. This goal is significantly aided by consolidating and critically considering the findings of early workers, as well as the continuum of evolving prior knowledge concerning relevant aspects of this reflex. It is for this purpose that the present narrative review is intended.

## Review

Methods

This review is derived from information obtained from an analysis of published material secured via literature searches of four major electronic databases and, in part, based on the author’s experience with the method. The databases searched were PubMed, Web of Science, EMBASE, and CINAHL Complete. The literature search covered all years up to 2026 and used the primary search string: (“inspiratory gasp” OR “inspiratory gasp vascular reflex” OR “inspiratory gasp reflex”). Studies considered relevant for inclusion were those that evaluated the IGVR in healthy persons or persons with underlying conditions and were published in English or French. The combined number of titles identified was 236, of which 155 were duplicates, yielding 81 titles of potential relevance. Abstracts of these were screened, and some were deemed irrelevant primarily because they did not address the vascular response in their studies. Full texts of the remainder were read in detail and are referenced in this review. Searches were completed on February 2, 2026.

Early pioneering studies

In 1936, Bolton et al. [7] reported their observations on 20 healthy subjects of the decrease in the volume of hand and foot digits two to three seconds after a sigh. This observation led them to explore the process in detail by replicating the sigh using intentional IGs. From these experiments on the limited number of healthy subjects and a few with various neurological conditions, they concluded that this reflex depends on an intact peripheral sympathetic system, as the vasoconstrictor response was absent in the digits of limbs that were either denervated or sympathectomized. They also provided experimental evidence that the primary stimulus for the response was the inspiratory phase of the IG, and the reflex itself depended on thoracic expansion. This latter observation was based on experiments in which thoracic expansion was largely prevented during breathing, and an IG did not cause the expected reduction in digit volume. A few years later, in 1939, Mulinos and Shulman studied this issue using multiple experimental modalities [8]. These included measuring hand blood flow using plethysmography and microscopically visualizing nail-fold capillary red blood cell flux. The blood flow measurements showed that a single IG reduced blood flow by an average of 83% in 48 subjects, with 2/3 of them demonstrating blood flow reduction or complete cessation, with the reduction or stoppage lasting two to eight seconds. Questions that remained unanswered were (1) what accounts for the variability in this duration among subjects and (2) what accounts for the 1/3 of subjects in whom no or only a minimal response was observed.

When the nailfold capillaries were observed in the presence of a single IG, the red blood cell flux came to a complete standstill in 83% of the 30 healthy subjects studied. The stoppage was reported to last three to five seconds, with a return to a normal flux rate approximately five to 15 seconds after the initial IG. That these responses were due to arteriolar vasoconstriction rather than larger diameter arterial or venous constriction was inferred from the following observations after a blood pressure cuff around the upper arm was inflated to 250 mmHg. Under this now-zero capillary red cell flux, an IG caused forward movement of the previously stationary red cells. This observation seemed to rule out constriction of vessels that were distal to the capillaries as the vascular change triggered by the IG. It also seems to rule in a proximal constriction close to the start of the capillary network. Hence, the concept of an arteriolar-dependent reflex was proposed. However, at that time, it was unclear whether any of the observed effects could be at least partly attributable to an IG-induced decrease in arterial blood pressure. Some clarification was provided by studies conducted by Gilliatt, as reported in 1948 [9,10]. He demonstrated variable blood pressure changes in response to IGs in healthy people and concluded the reflex was not a reduced blood pressure-triggered event. Furthermore, based on somewhat limited data, they suggested that the magnitude of the vasoconstriction was influenced by the initial lung volume at the time of IG.

In the same year, Gilliatt et al. sought to identify the afferent neural pathway associated with the IGVR by evaluating 10 patients with spinal cord lesions at C6-T11 [10]. Their approach was to measure finger and toe vasoconstriction responses to an IG. Based on their observations, they concluded that the IGVR was a purely spinal reflex originating from chest wall receptors and that the afferent fibers associated with it enter the spinal cord primarily in the upper thoracic region, with no participation of higher centers needed, although some doubt remains about the full afferent pathway, as De Lalla reported [11] that he could induce the reflex with either deep inspiration or negative-pressure breathing without chest-wall expansion. He argued that an increase in the intrathoracic venous stretch was the main initiating factor. The full afferent and efferent pathways responsible for the IGVR remain incompletely described.

Healthy people’s IGVR values

Finger Pulp Assessments

One of the earliest applications of LDF to systematically study aspects of the IGVR was reported in 1983 by Low et al. [12]. They measured IGVR with the LDF probe on the pulp of the index finger and the great toe of 63 healthy control subjects (29 male). They compared the IGVR with other interventions designed to elicit peripheral vasoconstriction. Tests were performed in a room maintained at approximately 26.7 °C, with the hand and foot warmed to between 34 °C and 35 °C. The IG was deep and rapid, with a 10-second breath-hold. Finger responses had a median reduction of 45% in males and 40% in females (p = 0.048). For toes, both sexes had the same 45% reduction. In 10 subjects, a repeat finger assessment on a different day yielded a coefficient of determination (R²) of 65%. It is important to note that these results were based on a wide age-group range (10-19 to 60-69 years) and that only a few participants (three to six) were in each group. There was also strict adherence to providing sufficient control of room and skin temperature to minimize excessive sympathetic tone to the digit arterioles prior to starting the IG. Building on these findings, Kahn et al. in 1988 [13,14] measured LDF at the fingertips of 20 young adults (26.0 ± 4.7 years) on three separate days and assessed IGVR as the percentage decrease in LDF accompanying a single IG. For this group, the IGVR was 77 ± 8%. These reductions were considerably greater than those reported by Low et al. [12]. The large difference between these percentage reductions is unlikely to be explained by subjects’ age, since Kahn et al. [13] reported a 71 ± 11% reduction in an older group (50.9 ± 15.8 years) of 10 healthy individuals, although in 10 age-matched diabetic patients with autonomic neuropathy, the LDF reduction was only 24 ± 15%, which was statistically lower than in the age-matched group (p < 0.005). It should be noted that the repeatability of the assessment was facilitated by producing a stable pre-IG vasodilated state in the examined digit by placing the other arm in water heated to about 43 °C. This procedure may not be necessary using current technology, since simultaneous local heating and LDF measurement is now possible [5]. One possible explanation for the difference in response magnitudes is that the high local and ambient temperatures used by Low et al. [12] may have blunted the impact of the sympathetic drive associated with the IGVR.

Further assessments of the IGVR were carried out by Feger and Braune in 2005 [14], who compared IGVR with other modalities in 60 healthy volunteers aged 20-78 years, including 28 females and 32 males. To help ensure sufficient initial vasodilation, subjects were evaluated in the supine position on a heating pad set to 36 °C while wearing gloves with the fingertips exposed. LDF was measured at the pulp of the index finger of both hands. Considering the entire group, they reported a median percentage decrease of approximately 71% for males and 56% for females (p < 0.01), with 1-2% differences between hands within groups. The median latency, measured from the start of the IG to the onset of the vasoconstriction, was reported as 3.6-3.8 seconds with no difference between genders, and the time to the minimum LDF value was 4.6-5.2 seconds, depending on the hand measured and gender. As previously noted, they also reported an age-dependent decrease in IGVR in both genders.

Given the reported variability in responses even among healthy subjects, this variability must be considered when comparing populations. It may be that the IGVR is best suited to assess longitudinal changes in the same individuals and to evaluate the effects of interventions targeting the vasculature and its sympathetic control. 

Finger Dorsum Assessment

**Figure 3:**
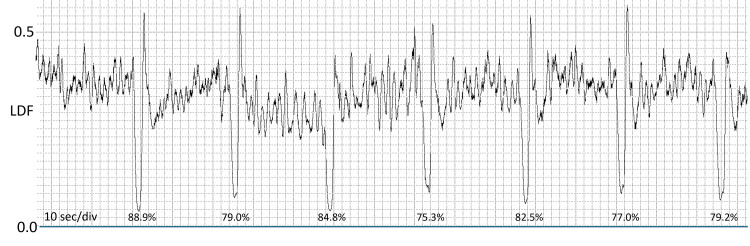
Example of sequential IGVR obtained on the finger dorsum The figure shows seven of 21 sequential IGVRs obtained from the dorsal aspect of the middle finger of a healthy subject. The percentages indicated are based on the %LDF reduction triggered by the IG. IG, inspiratory gasp; IGVR, inspiratory gasp vascular reflex; LDF, laser Doppler flowmetry This previously unpublished figure is provided courtesy of Harvey N. Mayrovitz.

Studies previously mentioned using finger LDF to assess the IGVR measured responses in the finger pulp, which is richly endowed with temperature-dependent arteriovenous anastomoses (AVAs). The response of relatively free AVA finger skin had not been determined, and in 2002, Mayrovitz and Groseclose investigated it [15]. In addition, they evaluated the sequential stability of the responses by having 28 healthy subjects (14 female) produce 21 sequential IGs, with each IG separated by two minutes from the previous one. They found that the overall average LDF reduction compared to the prior two-minute average was 72.2 ± 16.7%, with male reductions tending to be greater than those of females (77.1 ± 18.3 vs. 67.1 ± 14.3%, p = 0.062). These findings indicate that dorsal skin responses are similar to those obtained on skin with AVAs and that dorsal skin is an appropriate target for assessing IGVR. They also established that the sequential variability in response magnitudes is significantly less than inter-subject variability. The magnitude of these variations suggests that the IGVR is suitable for assessing longitudinal or interventional-induced changes within a subject but less suitable for comparing group-level differences in responses. One interesting but unexplained outcome was the presence of a large hyperemic response immediately following the IG. This overshoot, as well as an example of the sequential IG responses, is illustrated in Figure 3. In this figure, the percentage reductions in LDF are calculated as the percentage change relative to the average LDF of the immediately preceding minute.

**Figure 4:**
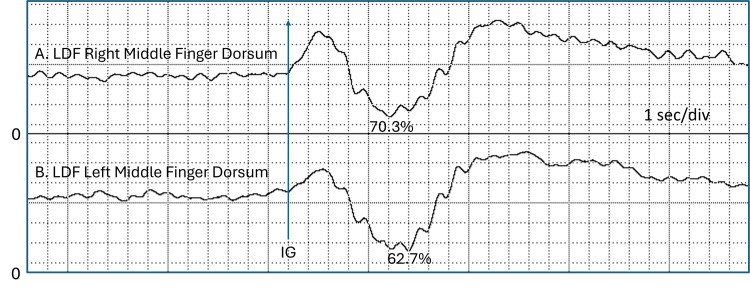
Example of bilateral responses to an IG Response to a single IG as measured on the middle finger dorsum of the right and left hands of a right-handed subject. The percentages indicated are based on the %LDF reduction triggered by the IG. IG, inspiratory gasp; LDF, laser Doppler flowmetry This previously unpublished figure is provided courtesy of Harvey N. Mayrovitz.

Potential response differences between hands have clinical importance, affecting procedures that use IGVR to evaluate suspected unilateral sympathetic dysfunction or to test the effectiveness of physiological interventions applied to a single limb of a pair. This aspect was specifically investigated by Mayrovitz and Groseclose [16]. LDF was measured on the middle finger dorsum of both hands during three sequential IGs separated by three minutes in 30 seated healthy subjects (15 females). IGVR was determined as the percentage decrease in LDF, considering the biological zero (BZ) obtained with LDF measurements using a compression cuff at the base of the fingers. The needed BZ correction accounted for approximately 3% of the response. The main results indicate an overall IGVR of 80.6 ± 11.4%, with no statistically significant difference between hands or genders, although males tended to have greater IG-induced LDF reductions than females (83.4 ± 11.4% vs. 78.7 ± 11.7%). Figure 4 illustrates a sample response of such bilateral measurements in response to a single IG. There is usually some differential in the percentage reduction between corresponding fingers on paired hands, with a reported maximum differential of 9.6 ± 8.5% [16]. The significance of such a difference becomes apparent when evaluating the presence of unilateral dysfunction. Based on the normal side-to-side variations above, a two-SD criterion would mean that a side-to-side differential of 26% or greater would be required to detect dysfunction. However, when using the IGVR to test the effect of an intervention in a normally functioning individual, a minimal detectable change of 10.1% has been reported, which is based on the upper limit of the 95% CI at a power of 0.9 [17].

Use of PPG to assess IGVR

The PPG signal contains time-varying pulsations caused by changes in blood volume, primarily due to cardiac-related arterial blood flow pulses. These pulsations are well seen in Figure 1 and Figure 2. The method’s first use on skin was reported by Hertzman in 1938 [18], and applications for assessing the IGVR are attributed to Barron et al. [19]. In addition to the volume changes due to the cardiac pulse, the average of the PPG signal depends on the total red cell volume within the measured skin tissue [20]. Thus, in a situation where pulsatile blood flow has ceased or is very small, the PPG signal will be zero or very small, but it will still have a nonzero average. This average value has been called the DC value, analogous to the electrical term “direct current,” and the alternating pulses are referred to as the AC component by Barron et al. [19]. They measured the reduction in the DC signal associated with an IG in 25 healthy young adults and reported substantial variability in the response; therefore, they concluded that an abnormal response could be identified only as consistently absent in patients with suspected neurological defects.

**Figure 5:**
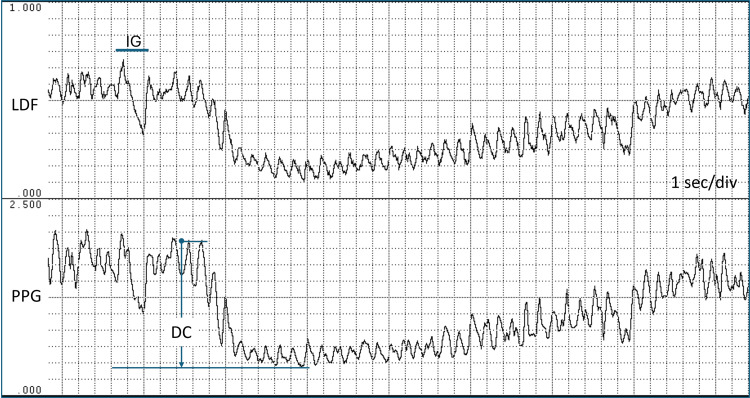
Assessment of the PPG change The LDF signal is measured on the ring finger of the left hand and the PPG on the index finger of the same hand. The DC value is the parameter measured from the peak of the pre-IG signal to the minimum value during the IGVR. The AC value is the amplitude of the cardiac pulsations prior to the decrease. For this example, the DC/AC ratio is 2.86. The corresponding maximum decrease in LDF associated with the IG is 83.6%. IG, inspiratory gasp; IGVR, inspiratory gasp vascular reflex; LDF, laser Doppler flowmetry; PPG, photoplethysmography This previously unpublished figure is provided courtesy of Harvey N. Mayrovitz.

The use of the DC PPG was extended by Allen et al., who examined both LDF and PPG to assess the vascular response to an IG and the repeatability of the response in healthy participants [21]. They evaluated responses by measuring LDF, PPG, and skin temperature (TSK) on the pulp of three adjacent fingers in 15 seated adults with an average age of 33 ± 10 years. The testing was conducted in a room maintained at 26 °C, with the subjects’ contralateral hand immersed in water at 40-42 °C. Three IGs, each lasting approximately two seconds and separated by 90 seconds, were performed, each starting from a normal eupneic end-expiration. This test sequence was repeated within four weeks. The median LDF percentage reduction was reported as 92.7% with a 95% CI of 53.2-98.4%. This value is somewhat higher than prior reports but may be attributable to the combined effects of elevated room temperature and hot-water hand immersion, which together reduce resting arteriolar tone. To evaluate the PPG response to an IG, they introduced the DC/AC parameter. The DC value was defined as the difference between the maximum PPG signal immediately preceding the IG and the minimum PPG value during the response. The AC value was defined as the peak-to-peak value of the PPG signal just prior to the IG. For the 15 healthy subjects, they reported a median DC/AC ratio of 2.6 (95% CI: 1.6-4.2) and, in a later paper, suggested the IG test as possibly useful for patients with Raynaud’s syndrome and complex regional dystrophy [3]. Figure 5 from the present author illustrates the DC measurement using a PPG measured on the index finger. The calculated DC/AC ratio is 2.86, and the corresponding decrease in LDF is 82.3%.

Using finger PPGs, Mueck-Weymann and Rauh investigated the influence of preceding IGs on the subsequent amplitude of following IGs [22]. Their approach was to evaluate the response amplitudes of PPG signals across five consecutive IGs, with delays between IGs ranging from 20 to 180 seconds. It is difficult to interpret their findings fully because they based the outcome on changes relative to a single initial IG and were unclear about the precise method used to calculate these changes. Additionally, their testing was conducted in a room at a particularly low temperature of 21-22 °C, which would affect the degree of basal sympathetic tone in the skin arterioles. With these provisos in mind, they reported that about half (56%) of the 25 young adults evaluated reached their maximum IGVR on the first IG. Based on simultaneous measurements of the sympathetic skin response (SSR) and the IGVR via PPG in 30 healthy subjects, it was shown that both occur during deep inspiration and that a one-minute delay is adequate for evaluating the amplitudes of sequential IGVR [23]. However, a two-minute delay appears preferable in the current author’s experience.

Site of measurement as a factor

In 1993, Valley et al. evaluated the IGVR in 10 healthy volunteers (five female) as a prelude for its potential use in epidural anesthesia [24]. They measured LDF at the thenar eminence, with skin temperature maintained at 36 °C, while the subject performed an IG and held their breath for four seconds. The IG-induced LDF percentage reduction was 54 ± 6 %. This value is lower than the 70-80% reductions reported previously, possibly because of the differential reactivity of arterioles within the thenar eminence as compared with the finger pulp. This group then used the IGVR to assess its utility in 40 patients undergoing epidural anesthesia, with IGVR measurements obtained on the foot dorsum [25]. Their data suggested that a negative IGVR to a 5 ml anesthetic test dose would indicate a proper catheter placement for anesthetic drug delivery.

All fingers of both hands were assessed using LDF in 47 healthy individuals who served as a control group to evaluate neural dysfunction in individuals in contact with patients with leprosy [26,27]. The data from the 1997 study indicated some finger-dependent variations in IGVR, ranging from an IGVR value of approximately 62% at the thumb to 72% at the pinky and ring fingers. An overall value for all fingers was reported as 66.8 ± 7.8%. These were significantly different from those measured in 89 leprosy patients [27] and in 24 asymptomatic persons living with persons with active leprosy (57.8 ± 14.6, p = 0.001) [26]. IGVR at the big toe for controls averaged 59% ± 17% and did not differ from that of household contacts. Netten et al. [28] also reported the impact of IGs measured on the pulp of the great toe with the toe unheated and, in 10 participants, locally heated to 36 °C. Among other tests, the IG response was determined once with the participant supine and then repeated approximately five minutes later. The percentage LDF reductions reported for these healthy young subjects were 31.8 ± 14.8% and 25.8 ± 14.8% for the sequential measurements. These reductions are considerably lower than the approximately 70% reductions reported by others for IG-related finger responses. However, toe measurements in elderly subjects without peripheral arterial disease have been reported to yield similar IGVR values [29]. This reviewer suspects that the supine position being used limited the magnitude of the inspired volume and, to some extent, affected the results. However, it is not clear if, in general, the lower extremity reflex responses are in principle different than upper extremity responses.

Age and vital capacity as factors

In 1995, the possible dependence of the IGVR on gender and age was the initial target of Lau et al. [30]. They used fingertip LDF changes to assess the vascular response to IG and quantified them as a percentage change from a stable baseline, as Kahn et al. [13] had done four years earlier. However, in the latter study, each participant’s vital capacity was also measured. This was an important addition to the study protocol because the magnitude of the IG response, which was greater in young males than in either young females or older males, was directly related to the participant’s vital capacity. Furthermore, when five young males varied their inspired volume during IGs, the percentage reduction in LDF was linearly related to the inspired volume expressed as a percentage of each individual’s vital capacity. Importantly, from these measurements, it was found that an inspiratory volume greater than approximately 30-50% of the individual’s vital capacity was needed to evoke a nonzero vascular response. The following year (1996), Liu et al. [31] extended this work to determine the impact of cigarette smoking on the vascular response to IGs using the same basic protocol. Their results were consistent with the important (though not the only) role of vital capacity in influencing the IG vascular response. Rauh et al. reported a 70% reduction as a threshold for a precipitous decrease in oxygen saturation when assessed on the finger during IGs in healthy young adults [32].

Based on prior observations of a relationship between the IG-induced LDF reduction and inspiratory volume, a study was undertaken to determine the impact of controlling inspiratory volume during IG using spirometry [33]. In this study, 19 healthy individuals under the age of 40 were evaluated while supine, with LDF measured on the pulp of the great toe of one foot. Among the goals was to determine the effect of inspiration rate and volume on the resultant decrease in LDF flow. A total of 13 sequential IGs were used to vary the parameters being assessed. By having participants inspire to their maximum volume in two, five, or 10 seconds, it was determined that the higher inspiratory velocity resulted in the largest LDF decrease, which averaged 70% with a range of 19-90%, as compared to a 10-second inspiration, which yielded a 62% reduction with a range of 39-80% (p < 0.05). The time to reach the minimum LDF value was shorter at the faster inspiration rate, 12 seconds (7-16) vs. 14 seconds (3-19), p < 0.05. Despite differences in the percentage reduction, depending on the point in the respiratory cycle at which the IG was initiated, it was concluded that, for testing purposes, an IG taken without specific controls on inspiratory volume, with breath held for 10 seconds, is adequate and valid.

Clinical assessments 

Neural Function

An early attempt to use the IGVR for clinical assessment of neural function was made by Schürmann et al. [34]. They used the percentage decrease in LDF, as others had done, as an indicator in 15 patients with reflex sympathetic dystrophy (RSD), compared with 15 age-matched controls and 52 healthy individuals spanning a wide age range (17-75 years). LDF was measured on the middle finger pulp, to which an LDF probe and a thermostatic heater were attached, set to 40 °C, and maintained at 40 °C. A heating pad applied to the subject’s back was also used to promote generalized vasodilation. LDF percent decreases for 27 males and 25 females were reported as 61 ± 14% and 70 ± 19%, respectively. IG Data comparing RSD patients with age-matched controls showed a markedly reduced IGVR (11 ± 7% vs 55 ± 18%, p < 0.001). Measurements from four healthy adults over 10 days were used to assess the reproducibility of the IG-induced LDF reduction, but the subjects’ ages were not reported. It should be noted that in this series and most others previously described, the BZ of the LDF signal has not been accounted for. The BZ is the residual signal present even when zero flow is occurring [35]. If this were taken into account, the percentage reduction would be slightly lower than reported. However, based on data from 52 healthy subjects, the overall reference range, using the mean ± SD, would be 47-75% for males and 51-89% for females.

Further studies of patients with RSD were undertaken by Ide et al. [36]. They measured IGVR values in the fingers of both hands and determined the inter-hand IGVR ratio in 20 patients with unilateral RSD and compared these values with those in a control group of 10 healthy subjects. A significant difference was found between controls and patients with stage II RSD (n = 9; p = 0.019), but not for controls and patients with stage I RSD (n = 11).

To assess the efficacy of an interscalene brachial plexus block in patients recovering from acromioplasty, the percentage decrease in finger LDF induced by IG was evaluated in eight patients at zero, two, and four hours post-surgery [37]. In the unblocked arm, the LDF reductions at these times post-surgery were 58%, 47%, and 56%, respectively. For the blocked arm, no significant percentage LDF reduction was reported at any of the measured time points. However, a review of their Figure 4 data indicates that, even in the blocked arm, LDF decreased by approximately 15-25%. Thus, the LDF reduction in the blocked arm is attenuated but not zero. However, the authors suggest that this method is useful for assessing the effectiveness of an interscalene blockade. A similar use of the IG test has been suggested for assessing ulnar nerve blockade [38]. This was based on LDF fingertip measurements in nine healthy subjects before and after ulnar nerve block, in which a 48.2 ± 5.3% LDF reduction was decreased to 3.1 ± 0.9% after blockade. Furthermore, the IG test has been used to assess sympathetic vasoconstrictor fiber function in 40 patients with peripheral neuropathy compared with healthy controls [39]. The percentage decrease in index finger LDF was calculated for patients whose baseline LDF exceeded a threshold, thereby excluding responses from patients with very high resting sympathetic drive. This resulted in the inclusion of 37 patients, and their responses were compared with those of 37 age-matched controls (19 women and 18 men). Controls had a 68.0 ± 15.5% reduction in LDF, compared with 50.1 ± 19.1% in patients (p < 0.001). The subject’s position was not specified in the study description, nor was the BZ accounted for. However, the authors concluded that the test is a useful, noninvasive tool for detecting sympathetic small-fiber dysfunction.

Diabetes

Barron et al. evaluated 30 patients who had diabetes mellitus (DM) for at least 10 years using DC changes in PPG recorded on the finger [19]. Of the 30, 14 (47%) had no measurable IGVR response. The absence of a response was observed in patients with some degree of peripheral neuropathy. Abbot et al., using LDF changes measured at the fingertip, reported normal IGVR in patients with uncomplicated DM but significantly reduced IGVR in patients with diabetic neuropathy [40]. Khan et al. [41] evaluated 10 DM patients with autonomic neuropathy and found significantly reduced IGVR as compared to 10 age-matched controls (15 ± 12% vs. 71 ± 11%, p < 0.005). Similar findings were reported by Netten et al. for IGVR when LDF was measured on the great toe of 20 DM patients with neuropathy and 20 without [42]. They found that the uncomplicated DM patient’s IGVR did not differ from controls (48.2 ± 4.1% vs. 49.0 ± 3.8%), but those with neuropathy had IGVRs that were significantly reduced (31.4 ± 4.6%, p < 0.05). In contrast, Wilson et al., who also used finger LDF measures, reported no difference in IGVR between 29 patients with DM and 15 controls [43]. To investigate painful neuropathy in patients with DM, Quattrini et al. evaluated eight patients with neuropathic pain, 10 persons with DM who had neuropathy but no pain, 10 persons with DM free of neuropathy, and 10 controls [44]. They measured IGVR on the great toe pulp using LDF with each subject supine in a room at 22 °C. Results indicated a mean IGVR of approximately 46% across all groups, except in those with pain, who had a mean IGVR of 14 ± 9%, which was statistically lower than for all other groups.

Leprosy

Leprosy is associated with nerve damage in small myelinated and unmyelinated nerve fibers. Testing for early changes in IGVR has been undertaken by several groups. In the early 1990s, Abbot et al. used LDF finger assessments and reported significant reductions in IGVR among the leprosy patients studied [40,45,46]. Subsequent studies revealed reductions in finger IGVR even in people free of leprosy, but who were in contact with or lived with people with leprosy [26,27]. In 76 newly diagnosed patients with leprosy, the IGVR, as measured with LDF on the finger pulp, was reported to be abnormally low in 43% of the patients evaluated [47].

Complex Regional Pain Syndrome (CRPS)

The IGVR has been used to demonstrate the near absence of sympathetic response, measured with finger LDF, in a patient with unilateral CRPS-1 during the acute phase, whereas the contralateral limb had a near-normal LDF reduction [48]. Normalization of the IGVR was demonstrated to occur seven weeks after the initial onset. This suggests the potential use of the IGVR as a follow-up measure to track recovery. Other reports of the use of IGVR testing in CRPS, however, reported no side-to-side differences in IGVR when a group of 14 patients with CRPS-1 was similarly evaluated [49]. Thus, the utility of IGVR in this condition remains to be determined by additional research.

Erythromelalgia

Littleford et al. investigated the potential sympathetic component associated with erythromelalgia in 83 patients by measuring the IGVR in fingers and toes while subjects were in a semi-recumbent position, with one arm immersed in water heated to 44 °C [50]. To achieve a significant vasodilatory state, they also maintained the room at 28 °C. IGVR responses were significantly lower than those measured in a group of 30 age-matched healthy controls. For the controls, IGVR values were 70 ± 13% at the fingers and toes, whereas in patients, they were reduced to 39 ± 47% and 45 ± 54% at the toes and fingers, respectively. Despite the large SD, they report a significant difference between patients and controls (p < 0.001).

Arterial Disease

Nukada et al. evaluated IGVR in toes of 44 patients with varying degrees of peripheral arterial disease and compared these to 30 controls of similar ages [29]. In the absence of DM, the percentage reductions in LDF for PAD patients did not differ significantly from those of controls in the seventh or eighth decades. However, the reported values for the control group were themselves low, with seventh- and eighth-decade controls showing average LDF percentage reductions of 39.3% and 29.4%, respectively. This reviewer suspects that these lower values are in part attributable to the low average skin temperature reported for the toe (25.6 °C), in part to the subjects’ supine position at the time of evaluation, and to their age-related reduced vital capacity. However, these confounding variables do not dispute the fact that no statistically significant difference was detected between those with and without PAD.

Assessing Drug Effects

The vasoconstrictive effectiveness of infusing the rapid-acting alpha-adrenergic antagonist phentolamine was evaluated in 16 patients with chronic unilateral extremity pain using a low (n = 8) and higher (n = 16) dose and measuring the IGVR at the finger or toe of the affected and contralateral limb [51]. Prior to drug infusion, IGVR in both limbs was similar, ranging from 78.3 ± 4.4 in the nonpainful limb to 74.2 ± 3.6% in the painful limb. The low and high doses (0.5 mg/kg and 1.0 mg/kg) attenuated the IG-induced reduction in LDF by 14.1 ± 5.8% and 23.3 ± 5.7%, respectively, with no difference between the painful and nonpainful limbs.

Discussion

The overriding evidence from the literature indicates that the IGVR can provide useful information about the status of peripheral neural function. However, its application and interpretation may be deceptively simple. The measurement methodology is indeed straightforward, using either LDF or PPG changes induced by one or more IGs. However, measurement conditions contribute to variability in response magnitude, making it difficult to establish “normal” ranges. In some studies, great pains were taken to provide local and ambient conditions that favor reduced sympathetic tone in peripheral arterioles. This took the form of direct localized heating alone or combined with indirect heating via elevated room temperature, application of a heating pad, or placing the contralateral limb in heated water. The degree of pre-IG vascular tone reduction likely varied by method, thereby contributing to variability in the magnitude of the IGVR even in healthy people. Further contributing to the variability in the magnitude of the IGVR was its apparent dependence on a person’s vital capacity, which itself was age- and gender-dependent. Based on these reported findings, the present author’s opinion is that the best use of the IGVR is for assessing longitudinal changes in the same person under similar environmental and local conditions. Such longitudinal assessments could use the IGVR to evaluate the effects of a specific intervention thought to have neurovascular effects, delivered either acutely in a single application or sequentially over multiple applications over successive days or weeks. It could also be used to track temporal changes in a patient’s condition. However, its use in comparing neurovascular responses between two patient groups or between a patient and a control group is importantly affected by variability, since similar local and environmental conditions may not affect the groups equally. This is not to say that IGVR should not be used to compare groups; it is only to point out the confounding factors to be considered in such comparisons.

**Figure 6:**
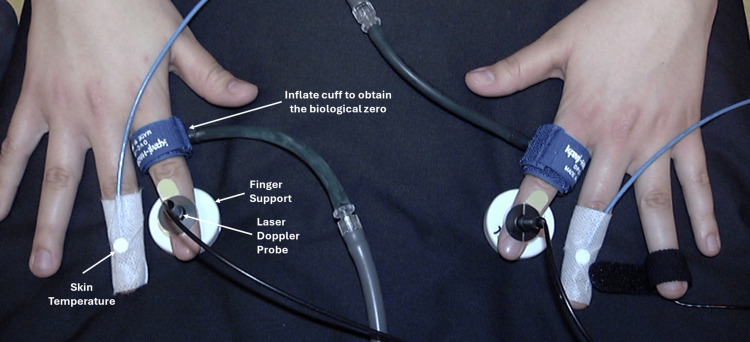
Setup for bilateral finger dorsum laser Doppler-determined IGVR The target fingers are supported for comfort on a surface that could be heated, if desired. Basal skin temperature may be recorded with a suitable thermistor on an adjacent finger, as shown. The cuff at the base of the fingers is inflated at the end of the experiment to record the LDF BZ. The BZ is the value of the laser Doppler output when blood flow is forced to zero by the cuff inflation. BZ, biological zero; IGVR, inspiratory gasp vascular reflex; LDF, laser Doppler flowmetry This previously unpublished figure is provided courtesy of Harvey N. Mayrovitz.

The literature does not fully clarify the most appropriate environmental and local conditions to be used when evaluating the IGVR. When evaluating the upper limb using fingers for LDF or PPG measurements or the hand for PPG measurements, the subject’s position (seated, supine, or semi-recumbent) affects the IGVR value. Of these three positions, the seated position offers greater freedom for the IG and is recommended to probably achieve the largest response and least variability. However, this has not been verified experimentally, and research into this aspect is warranted. Furthermore, the need to use extensive measures to heat the body, such as contralateral arm warming or heating pads, although sensible to blunt vascular tone, is unverified as a way to improve variability in the IGVR among subjects within groups. The use of local skin heating in combination with LDF may be sufficient for many purposes. This gap in our understanding is also ripe for further research. When LDF is used as an assessment, a choice needs to be made between the finger pulp and the dorsum, since both sites have been used successfully. From the subject’s or patient’s standpoint, the hand position for dorsum measurements is usually more comfortable, and placement of the LDF probes is easier. Patient comfort may promote more consistent IGVR responses. An example of a bilateral finger dorsum LDF measurement setup for obtaining IGVRs is illustrated in Figure 6.

When the focus is on lower extremity neurovascular responses, a suitable subject position is either supine or semi-recumbent. From the viewpoint of having the least resistance to thorax expansion during the IG, it would appear that the semi-recumbent position should be adopted as a standard when IGVR assessments are done on the toes or foot dorsum. Most studies evaluating the lower extremities have used LDF measurements on the great toe pulp. The choice of this digit is in part based on its larger surface area, which accommodates the probe. However, because of the near-vertical position of the toe, in a semi-recumbent position, it is difficult to maintain a uniform probe-skin perpendicular pressure. Such variations may contribute to variability in sequential responses. Efforts to minimize this effect should be considered, perhaps by standardizing measurements to the foot dorsum, which has a more solid foundation than the toe. Comparisons between IGVR responses on the foot dorsum and the toe are needed as future research targets.

## Conclusions

The most useful application of the IGVR is to assess changes in peripheral neurovascular function in individuals following interventions or treatments that target such functions or to track longitudinal changes. Its use in discriminating between patient groups with various peripheral neurovascular dysfunction and controls is limited by the absence of suitable normal reference ranges and standardized measurement protocols. However, when confounding factors are considered and group differences are sufficiently large, the IGVR can be a useful test. Future research should focus on standardization, site optimization, positional effects, and establishing reference ranges, especially for the lower extremities.
